# Hematopoietic Stem Cell Transplantation in Primary Immunodeficiency Patients in the Black Sea Region of Turkey

**DOI:** 10.4274/tjh.2016.0477

**Published:** 2017-12-01

**Authors:** Alişan Yıldıran, Mehmet Halil Çeliksoy, Stephan Borte, Şükrü Nail Güner, Murat Elli, Tunç Fışgın, Emel Özyürek, Recep Sancak, Gönül Oğur

**Affiliations:** 1 Ondokuz Mayıs University Faculty of Medicine, Department of Pediatric Allergy and Immunology, Samsun, Turkey; 2 Leipzig University, Translational Centre for Regenerative Medicine, Leipzig, Germany; 3 Ondokuz Mayıs University Faculty of Medicine, Department of Pediatric Hematology and Oncology, Samsun, Turkey; 4 Ondokuz Mayıs University Faculty of Medicine, Department of Pediatric Genetic, Samsun, Turkey

**Keywords:** Hematopoietic stem cell, Transplantation, Children, Immunodeficiency

## Abstract

Hematopoietic stem cell transplantation is a promising curative therapy for many combined primary immunodeficiencies and phagocytic disorders. We retrospectively reviewed pediatric cases of patients diagnosed with primary immunodeficiencies and scheduled for hematopoietic stem cell transplantation. We identified 22 patients (median age, 6 months; age range, 1 month to 10 years) with various diagnoses who received hematopoietic stem cell transplantation. The patient diagnoses included severe combined immunodeficiency (n=11), Chediak-Higashi syndrome (n=2), leukocyte adhesion deficiency (n=2), MHC class 2 deficiency (n=2), chronic granulomatous syndrome (n=2), hemophagocytic lymphohistiocytosis (n=1), Wiskott-Aldrich syndrome (n=1), and Omenn syndrome (n=1). Of the 22 patients, 7 received human leukocyte antigen-matched related hematopoietic stem cell transplantation, 12 received haploidentical hematopoietic stem cell transplantation, and 2 received matched unrelated hematopoietic stem cell transplantation. The results showed that 5 patients had graft failure. Fourteen patients survived, yielding an overall survival rate of 67%. Screening newborn infants for primary immunodeficiency diseases may result in timely administration of hematopoietic stem cell transplantation.

## Abstract (tr)

Birçok kombine primer immün yetmezlik ve fagositer bozukluk için hematopoetik kök hücre nakli küratif bir tedavidir. Bu çalışmada, primer immün yetmezlik tanısı alan ve hematopoetik kök hücre nakli yapılan hastaları retrospektif olarak inceledik. Yirmi iki hasta belirlendi. Hastaların hematopoetik kök hücre nakli sırasındaki ortanca yaşları 6 ay (minimum-maksimum: 1 ay-10 yaş) idi. Hastaların tanıları ağır kombine immün yetmezlik (n=11), Cheidak Higashi sendromu (n=2), lökosit adezyon defekti (n=2), MHC sınıf-2 eksikliği (n=2), kronik granülomatoz hastalık (n=2), hemofagositik lenfohistiyositoz (n=1), Wiskott-Aldrich sendromu (n=1) ve Omenn’s sendromu (n=1) idi. Yedi hastaya tam insan lökosit antijen uyumlu, 12 hastaya yarı uyumlu ve 2 hastaya insan lökosit antijen uyumsuz vericiden hematopoetik kök hücre nakli yapıldı. Beş hasta da graft başarısız oldu. On dört hasta hayatta kaldı ve ortalama sağkalım %67 idi. Bu hastalık için yenidoğan taramaları yapılması ile hematopoetik kök hücre transplantasyonları zamanında yapılabilir.

## INTRODUCTION

Primary immunodeficiency (PID) disorders are a group of heterogeneous diseases, many of which are caused by monogenic defects, resulting in susceptibility to life-threatening infections, uncontrolled inflammation, or autoimmunity. In 1968, successful transplantation was performed in two patients, one with severe combined immunodeficiency (SCID) and one with Wiskott-Aldrich syndrome (WAS). These cases represented the first successful hematopoietic stem cell transplantation (HSCT) procedures, ushering in a new era of curative therapies for treating PID disorders [1 ,2 ,3]. To date, only one report has described HSCT therapies for PID disorders in Turkey [4]. The aim of this study was to retrospectively document all pediatric cases of patients diagnosed with PID disorders and considered for HSCT therapy at our pediatric transplantation center.

## MATERIALS AND METHODS

In total, 22 infants were diagnosed with PID; 19 of these patients underwent HSCT at the Ondokuz Mayıs University Faculty of Medicine, Department of Pediatrics, Pediatric Transplantation Unit, between June 2010 and December 2013. One patient died shortly after diagnosis.

Of the 22 patients, 11 were diagnosed with SCID, 2 with MHC class 2 deficiency, 2 with leukocyte adhesion deficiency (LAD), 2 with chronic granulomatous disease (CGD), 2 (siblings) with Chediak-Higashi syndrome (CHS), 1 with WAS, 1 with hemophagocytic lymphohistiocytosis, and 1 with Omenn syndrome (Tables 1 and 2). All patients met the European Society for Immunodeficiencies - Pan-American Group for Immunodeficiency diagnostic criteria for PID disease [5]. In terms of the phenotypic profiles of patients with SCID, seven displayed T-B-NK+ and four showed T-B+NK+ profiles. The molecular defects of two patients with SCID could not be determined. In total, eight patients with SCID and three without SCID underwent haploidentical CD34+ stem cell transplantation. Additionally, one patient with CGD and one patient with WAS underwent HSCT from matched unrelated donors at another center.

## RESULTS

### Patient Characteristics

The patients’ ages at diagnosis of SCID ranged from 2 to 8 months (median: 3 months). Parental consanguinity was determined in seven (64%) patients with SCID. Pneumonia and diarrhea were common complaints in patients with SCID. Parental consanguinity was determined in nine (82%) non-SCID patients. Two patients with SCID were referred by another center for HSCT 3 months after diagnosis. Only one SCID patient was found to be positive for cytomegalovirus antigenemia at the time of diagnosis; therefore, a conditioning regimen was not administered. No engrafted maternal T cells were detected in patients with SCID at the time of diagnosis. All patients with SCID were lymphopenic and had few T cells (CD3+ cells <30%).

The age of the non-SCID patients at the time of HSCT ranged from 3 to 120 months (median: 13 months). Failure to thrive was the most common complaint in non-SCID patients. Although parental consanguinity was determined in 64% of SCID and 82% of non-SCID patients, only six patients (27%) had matched related donors. The characteristics and transplantation data from SCID and non-SCID cases are shown in Tables 1 and 2.

### Complications

A common complication in our patients was graft failure (40%) that required repeated transplantations. Grade I-II acute graft-versus-host disease (GVHD) was observed in four patients (three SCID and one non-SCID) after HSCT. Outcomes and complications in the SCID and non-SCID cases are shown in Tables 3 and 4.

## DISCUSSION

Established in 2009, our bone morrow transplantation center was the first of its kind in the Black Sea Region of Turkey. We reviewed all pediatric patients diagnosed with PID who were scheduled to receive HSCT at Ondokuz Mayıs University between June 2010 and December 2013 (n=22). A similar recent study in two Balkan countries reported only 15 SCID cases during a 24-year period [6]. Cipe et al. [4] reported haploidentical HSCT in 18 patients in the capital city of Turkey during a 10-year period; however, many of these patients were from other regions of the country. Although four of our patients came from other regions (two with SCID, one with Omenn syndrome, and one with LAD), our patient numbers suggested that the prevalence of PID disorders should have been higher in the Black Sea Region of Turkey because of the high rate of consanguinity. Yorulmaz et al. [7] reported that the parental consanguinity rate was 37.5% in patients with PID; in another region of the country, these rates were 84%, 75%, and 73% in patients with SCID, phagocytic system defects, and common variable immune disease, respectively. As our prenatal consanguinity rates were lower than expected, we suggest that newborn screenings for PID disorders should be mandatory, at least in our region. In the near future, we plan to apply the screening method developed by Borte et al. [8], which includes a robust triplex polymerase chain reaction method for quantitation of T-cell receptor excision circles and κ-deleting recombination excision circles using single-punch Guthrie cards. We expect to identify patients with SCID, X-linked agammaglobulinemia, ataxia telangiectasia, Nijmegen breakage syndrome, and other severe immunodeficiency syndromes characterized by the absence of T or B cells with this method.

Cipe et al. [4] concluded that human leukocyte antigen (HLA)-haploidentical transplantation from parental donors represents a readily available treatment option, especially for patients with SCID, offering a high probability of cure. In our study, 12 patients received haploidentical HSCT, 7 received HLA-matched HSCT from related donors, and 2 received HLA-matched HSCT from unrelated donors. There are several concerns regarding the safety of haploidentical HSCT, as it may cause a delay in the successful outcome in patients with PID disorders. Our study and others [4,9] showed that T cell-depleted haploidentical HSCT is a life-saving treatment in patients with PID disorders.

In recent report from Jordan, a country that resembles Turkey socially, Amayiri et al. [10] concluded that delayed diagnosis (or referral) and reactivation of bacillus Calmette-Guerin (BCG) are unique challenges for patients with PID disorders. Similarly, delayed diagnosis is an important problem in our region because of the insufficient number of immunologists and lack of physician awareness about PID disorders [11]. BCG vaccine reactivation has an important effect on the prognosis of combined immunodeficiencies, but this vaccine also helps to identify interferon gamma/interleukin 2 axis defects with BCG-itis [12] and to determine T-lymphocyte function with a positive tuberculin (purified protein derivative) test.

Epidemiological studies in various countries have shown that X-linked common gamma-chain deficiency is the most common type of SCID, affecting almost half of all patients. In our patients with SCID, 55% had RAG1 and RAG2, 33% had JAK3, and 11% had IL2RG mutations. The present study showed that RAG mutations are more prevalent in SCID cases in Turkey than in Europe and the United States [13]. However, gamma-chain deficiencies are rare in the Greek population [14]. The higher incidence of RAG mutations in our region could be related to high parental consanguinity.

We used the CliniMACS method for efficient T-cell depletion prior to transplantation. After applying this method, we observed acute (18%-28%) and chronic (9%-18%) GVHD in the SCID and non-SCID cases. Our patients displayed low infection rates and BCG activation and less need for treatment in the pediatric intensive care unit (PICU), which could have been due to the use of prophylactic antituberculosis treatment.

A previous study addressing the outcomes of and mortality-related risk factors for pediatric patients with PID requiring PICU admission reported respiratory problems as the leading cause for hospital admission [15]. In our study, six patients required PICU admission, mostly due to severe infection and respiratory problems.

## CONCLUSION

This study showed that PID disorders are common and that the delayed diagnosis of such disorders is an important problem in the Black Sea Region of Turkey. Routine screening for these diseases should be performed in newborn infants.

## Figures and Tables

**Table 1:**
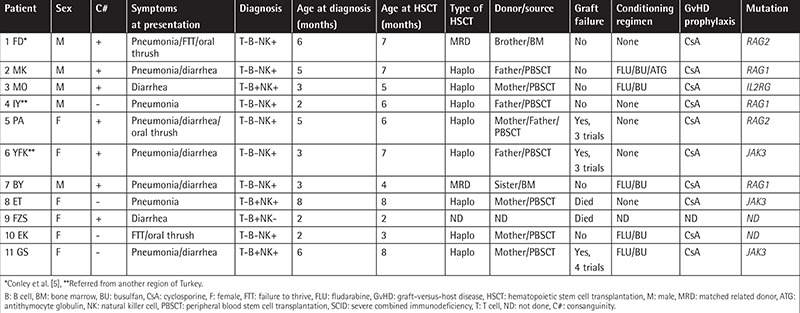
Characteristics and transplantation data of severe combined immunodeficiency patients.

**Table 2:**
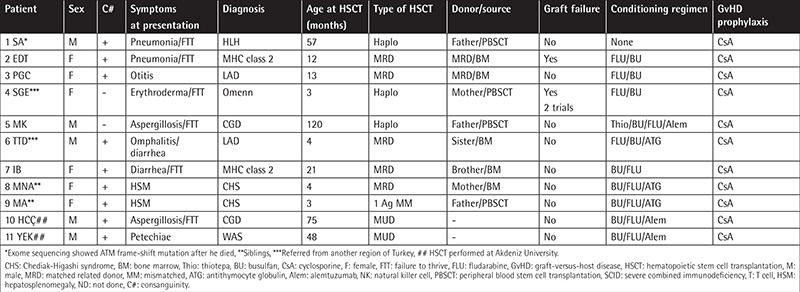
Characteristics and transplantation data of non-severe combined immunodeficiency patients.

**Table 3:**
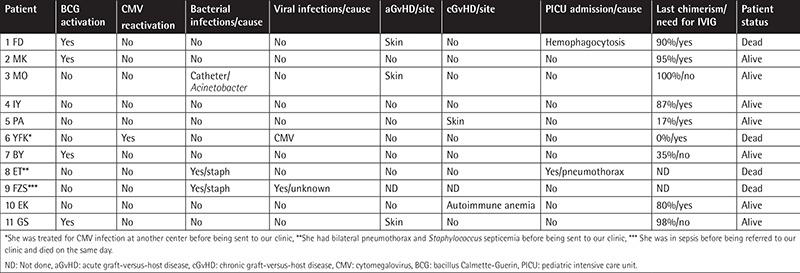
Outcomes and complications of severe combined immunodeficiency patients.

**Table 4:**
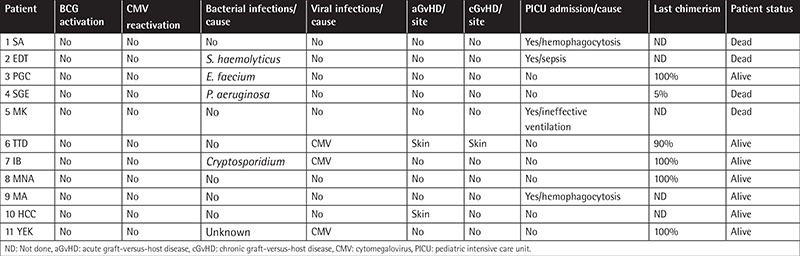
Outcomes and complications of non-SCID patients.
